# An overview of 18F-fluorodeoxyglucose positron emission tomography/computed tomography in giant cell arteritis

**DOI:** 10.3389/fmed.2024.1469964

**Published:** 2024-10-14

**Authors:** Thomas Thibault, Jean-Louis Alberini, Anne-Claire Billet, Hélène Greigert, André Ramon, Hervé Devilliers, Alexandre Cochet, Bernard Bonnotte, Maxime Samson

**Affiliations:** ^1^Department of Internal Medicine and Systemic Disease, Dijon University Hospital, Dijon, France; ^2^CHU Dijon Bourgogne, INSERM, Université de Bourgogne, CIC 1432, Module Épidémiologie Clinique, Dijon, France; ^3^Centre Georges Francois Leclerc, Service de Médecine Nucléaire, Dijon, France; ^4^Institut de Chimie Moléculaire de l’Université de Bourgogne, ICMUB UMR CNRS 6302, Université de Bourgogne, Dijon, France; ^5^Department of Internal Medicine, Hôpital Edouard Herriot, Hospices Civils de Lyon, Lyon, France; ^6^Department of Internal Medicine and Clinical Immunology, Dijon-Burgundy University Hospital, Dijon, France; ^7^Department of Vascular Medicine, Dijon University Hospital, Dijon, France; ^8^INSERM, EFS BFC, UMR1098, RIGHT Interactions Greffon-Hôte-Tumeur/Ingénierie Cellulaire et Génique, Université Bourgogne Franche-Comté, Dijon, France; ^9^Department of Rheumatology, Dijon University Hospital, Dijon, France

**Keywords:** giant cell arteritis, 18F-fluorodeoxyglucose positron emission tomography/computed tomography, diagnosis, monitoring, prognostic

## Abstract

PET/CT is an imaging modality that is increasingly being used to diagnose large-vessel vasculitis. In the case of giant cell arteritis, it was first used to demonstrate inflammation of the walls of large arterial trunks such as the aorta and its main branches, showing that aortic involvement is common in this vasculitis and associated with the occurrence of aortic complications such as aneurysms. More recently, with the advent of digital PET/CT, study of the cranial arteries (i.e., temporal, occipital, maxillary and vertebral arteries) has become possible, further increasing the diagnostic interest of this examination for the diagnosis of GCA. Despite these advantages, there are still limitations and questions regarding the use of PET/CT for the diagnosis and especially the follow-up of GCA. The aim of this review is to take stock of currently available data on the use of PET/CT for GCA diagnosis and follow-up.

## Introduction

Giant cell arteritis (GCA) is a large-vessel vasculitis (LVV) affecting people over the age of fifty (1, 2) and especially targets the aorta and branches of the external carotid arteries. The disease can cause vascular complications, particularly vision loss or stroke at diagnosis (3, 4), or aortic aneurysm in long-term follow-up (5).

GCA diagnosis is based on the combination of clinical signs of GCA with an increase in acute phase reactants (CRP, ESR) and evidence of vasculitis. Historically, temporal artery biopsy (TAB) was the gold standard to demonstrate granulomatous vasculitis (6). However, this examination is invasive (7) and lacks sensitivity (8), which led to vascular imaging’s growing role in confirming the diagnosis of GCA. Ultrasonography has been recommended by EULAR for several years as a first-line test to assess the temporal arteries in suspected GCA (9). This is also supported by the most recent EULAR guidelines, which state that ultrasonography of the temporal and axillary arteries is the first-line imaging test to be performed in this context (10). However, GCA does not always affect temporal arteries and sometimes targets large vessels, especially the aorta and its main branches, often in the upper limbs (11), and these areas are not readily accessible by ultrasound.

**Figure 1:**
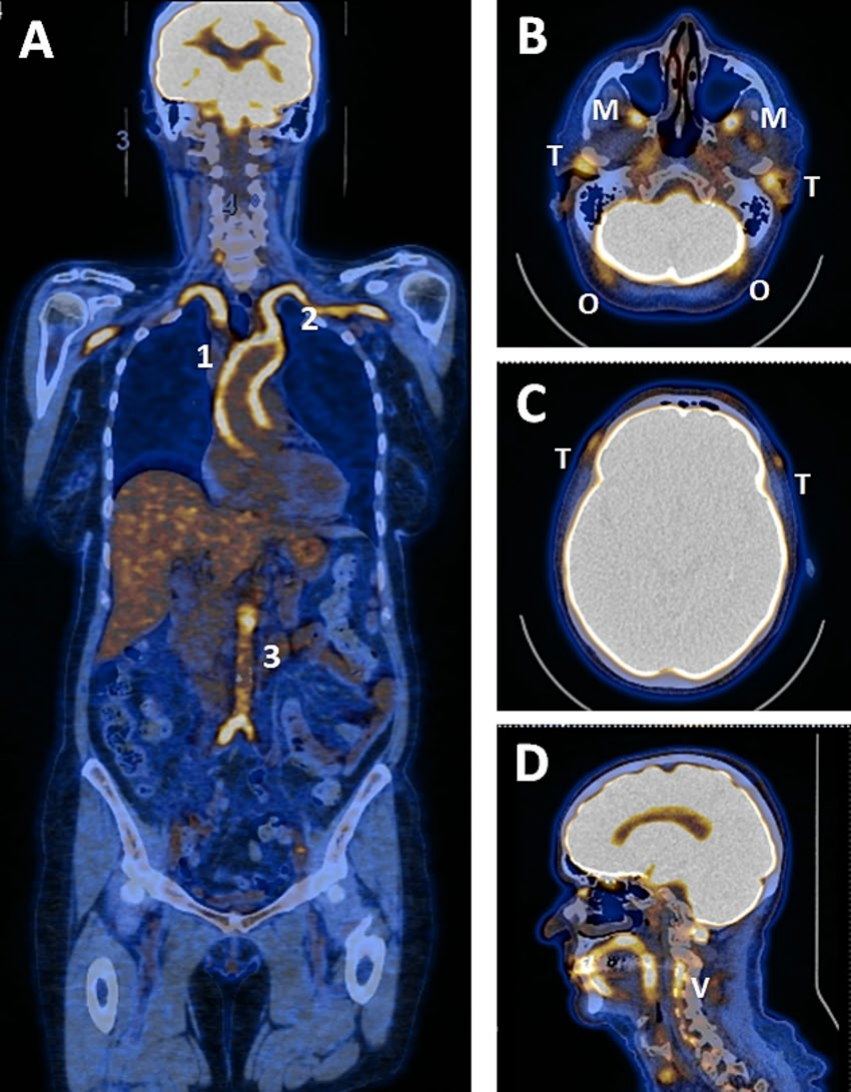
PET/CT study of a patient with GCA involving cephalic and large arteries. **(A)** study of large arteries showing grade 3 hypermetabolism of the ascending aorta (1), subclavian and axillary arteries (2) and from the abdominal aorta to the origin of the iliac arteries (3). **(B–D)** study of cephalic arteries showing significant hypermetabolism of the temporal (T), maxillary (M), occipital (O) and vertebral (V) arteries. **(C)** hypermetabolism of the frontal branch of the temporal artery (T).

Along this line, ^18^F-fluorodeoxyglucose positron emission tomography/computed tomography (PET/CT) has emerged as a very sensitive examination to detect LVV in GCA patients. More recently, newer generations of PET/CT have shown their good performance in demonstrating vasculitis of cranial arteries, including the temporal, occipital and maxillary arteries, allowing a more comprehensive assessment of vascular involvement (12–15), and are recognized in the new EULAR recommendations on imaging’s use in GCA (15) (Figure 1).

PET/CT is now widely used for GCA diagnosis, but there are still limitations to its application and interpretation. Indeed, PET/CT is highly sensitive to glucocorticoids and should therefore be performed before or as soon as possible after the start of treatment. A previous study showed that the hypermetabolic signal in large arteries decreased significantly after 72 h of treatment, meaning that this limit is often used as a quality criterion for PET/CT (16). However, it has been clearly demonstrated that arterial hypermetabolism can persist for many months after treatment begins, so this rule is not absolute.

PET/CT was first used to detect LVV but has since expanded beyond that setting and been evaluated in different contexts. Therefore, in addition to the diagnosis of GCA, PET/CT has been used for disease monitoring.

This report aims to provide an update on the performance of PET/CT in the diagnosis and monitoring of GCA.

## Generalities about PET/CT

The latest EULAR guidelines indicate that, in cases of high clinical suspicion and positive imaging, the diagnosis of GCA can be confirmed without additional tests, including TAB. The first-line imaging test to achieve this goal is ultrasonography of the temporal and axillary arteries. Furthermore, PET/CT remains the test of choice for evidencing LVV in extracranial arteries (aorta and proximal branches). PET/CT and MRI are also becoming an alternative to ultrasonography for the study of cranial arteries (10).

### Protocol procedure

With the aim of standardising procedures and optimising diagnostic accuracy, the recommendations reiterate good practice with regard to the protocol for performing PET/CT, including the acquisition of cranial artery imaging (Table 1) (15).

**Table 1:** Imaging modalities for PET/CT in LVV, according to EULAR recommendations (15).

⇒ Position of patient is supine, position of the arms should be arms down. ⇒ Body parts to include from top of head to at least mid-thigh, preferably to below the knees. ⇒ Blood glucose levels: preferred <7 mmol/L (126 mg/dL), <10 mmol/L (180 mg/dL) acceptable. ⇒ Interval between FDG infusion and image acquisition should be at least 60 min, preferably 90–120 min. ⇒ For evaluation of the cranial arteries, 5 min instead of 2–3 min acquisition time of the head should be used in cases of non-digital FDG-PET imaging. ⇒ Scoring of [18 F]-FDG-uptake: qualitative visual grading; if result is unclear, compare it to the liver background (grading 0–3). ⇒ Digital FDG-PET may be used in order to reduce imaging time, radiation dose and to improve the image quality. ⇒ FDG-PET is commonly combined with low-dose CT, optionally with CT-angiography (CTA). It can also be combined with MRI or MRA.

MRI, magnetic resonance imaging.

MRA, magnetic resonance angiography.

In particular, it is specified that the time between FDG infusion and image acquisition should be at least 60 min. Most studies on PET/CT in LVV have been conducted in these conditions. Delayed imaging at 3 h may provide a more detailed image of the arterial wall, mainly due to decreased blood pool activity, according to only one small prospective study of 23 patients with suspected LVV (17). However, there is little evidence to support a possible extension of this timeframe, in contrast to the recommended delay of 2 h for assessing the metabolic activity of atherosclerosis (18). Therefore, further studies extending the time between FDG infusion and imaging are needed to determine whether performance can be optimized in the diagnosis of GCA and LVV in general.

Before PET/CT, blood glucose levels should be closely monitored, especially in diabetic patients and after the introduction of glucocorticoids, as FDG uptake is reduced when serum glucose levels exceed 7 mmol/L (126 mg/dL) (19, 20).

According to Nielsen et al., diagnostic accuracy is not significantly affected when PET/CT is performed 3 days after the start of GC therapy, whereas it is significantly reduced when PET/CT is performed 10 days later (21). Therefore, PET/CT should be performed before or within this three-day period after starting GC to ensure good performance (16). The availability of PET/CT is one of the main limitations to its use in clinical practice, as it is often inappropriate to wait for a suspected GCA diagnosis before initiating glucocorticoid therapy due to the risk of ocular complications (16).

### PET/CT interpretation

There are several interpretation methods for assessing vascular hypermetabolism: the qualitative method, visual grading and semi-quantitative methods (19):

- The global qualitative method is still preferred in daily clinical practice due to the speed with which it can be initiated, as it is based on the clinician’s experience and overall visual assessment. PET/CT is defined as negative or positive according to the presence or absence of evidence of active LVV. However, particularly in the context of clinical research, standardization of interpretation and intra- and inter-observer reliability are not guaranteed with this approach.
- Qualitative visual grading is recommended in clinical practice when the result of the global qualitative method is unclear. Visual grading is based on comparing the intensity of vascular FDG uptake in each vascular segment with the background uptake in the liver. The resulting score ranges from 0 to 3: 0 = no FDG uptake (lower than the mediastinal blood pool); 1 = low-grade uptake (< liver uptake); 2 = intermediate-grade uptake (similar to liver uptake), 3 = high-grade uptake (> liver uptake). This score should be interpreted with caution due to frequent false positives related to atherosclerotic vascular uptake, particularly in the iliac and femoral arteries. According to guidelines, a score of 3 should be considered positive for active LVV and a score of 2 indicative of possible LVV (20). Lower scores are considered as negative for LVV. It should be noted that most PET/CT studies (extracranial and cranial PET/CT) use a visual grading of ≥2 to define PET/CT as positive.
- Semi-quantitative methods consist of directly measuring the SUVmax of vascular FDG uptake in each vascular segment. The target is defined by drawing a manually delineated volume of interest (VOI) that includes each vascular segment and avoids areas of atherosclerosis. Target-to-liver and target-to-blood pool ratios are calculated by dividing the SUVmax by liver or superior vena cava background, respectively. These ratios were proposed because the simple SUV metric does not seem relevant for initial diagnosis due to the high overlap between patients and controls (22), and the potential loss of specificity (23).

### Scores

Scores can be calculated by adding each vessel’s visual grading (from 0 to 3 points). Two scores are mainly used: TVS (Total Vascular Score) and PETVAS (PET Vascular Activity Score). TVS is defined by the addition of the Meller score (24), which is composed of 14 arterial territories ranging from 0 to 42 points, including the carotid arteries [*n* = 2], subclavian arteries [*n* = 2], axillary arteries [*n* = 2], ascending thoracic aorta [*n* = 1], aortic arch [*n* = 1], descending thoracic aorta [*n* = 1], abdominal aorta [*n* = 1], and the iliac arteries [*n* = 2] and femoral arteries [*n* = 2] (23). PETVAS includes 9 arterial territories, ranging from 0 to 27 points, including the ascending thoracic aorta [*n* = 1], aortic arch [*n* = 1], descending thoracic aorta [*n* = 1], abdominal aorta [*n* = 1], brachiocephalic trunk [*n* = 1], carotid arteries [*n* = 2] and subclavian arteries [*n* = 2] (25). Unlike TVS, PETVAS does not include the arteries of the lower limbs, where atheroma can interfere with interpretation of the uptake (24). The scores’ value is well correlated with vasculitis activity. Therefore, TVS and PETVAS are higher at GCA diagnosis than in treated GCA (26, 27). In addition, PETVAS is able to discriminate clinically active from inactive LVV with a sensitivity of 60% and specificity of 80% for a threshold of ≥10 points (28).

Dashora et al. (29) compared PETVAS with SUV semiquantitative metrics in 52 GCA and 43 Takayasu’s arteritis patients. Intra-rater reliability showed a better intraclass correlation (ICC) for the semiquantitative method [0.99 (range 0.98–1.00)] than for the visual grading by PETVAS [0.82 (range 0.56–0.93)]. When compared to physician assessment of clinical disease activity, the target-to-liver ratio had the highest area under the receiver operating characteristic curve (AUROC). The authors suggested that visual grading (such as PETVAS or TVS) should be used in clinical practice or observational studies when ease of interpretation is preferred, and SUV metrics should be used in randomized clinical trials or translational research when precision is mandatory.

## Diagnostic accuracy

Guidelines have specified that PET/CT can be used to detect mural inflammation or luminal changes affecting extracranial arteries in patients with suspected GCA (28).

Evaluating the diagnostic accuracy of PET/CT in GCA is challenging because no other available test, especially TAB, is a perfect gold standard due to lack of sensitivity (30). In some patients, only PET/CT can confirm the diagnosed GCA by showing high vascular uptake in cranial or extracranial arteries. To avoid this difficulty, recent studies have used a reference clinical diagnosis as a gold standard, i.e., a diagnosis maintained by the treating physician after 6 months of follow-up with no alternative found. These studies are compiled in the systematic review and metanalysis by Bosch et al. (31). Four studies with a low risk of bias (12, 15, 32, 33) which evaluated the diagnostic accuracy of PET/CT in suspected GCA compared with the reference clinical diagnosis were included. The four studies’ pooled results support high diagnostic accuracy (sensitivity 76% and specificity 95%). It should be noted that some of the studies include vascular FDG uptake in the cranial arteries to consider a positive PET/CT.

After evaluation of the diagnostic accuracy of PET/CT, there is a need for comparison of PET/CT with other imaging tests, particularly ultrasound of the temporal and axillary arteries. However, data about direct comparison between these two tests are lacking. Most published studies have included patients who had PET/CT or temporal ultrasound as the gold standard test (33–35). Therefore, the two tests cannot be compared. Other published trials evaluated the diagnostic accuracy of PET/CT and ultrasound using the clinical diagnosis confirmed after 6 months of follow-up as the gold standard. Unfortunately, at least one test was not performed in the whole population, which makes it difficult to draw firm conclusions in these studies (36, 37). Moreel et al. (38) published a systematic review and meta-analysis in 2023 with the aim of comparing PET/CT, ultrasound and MRI for the diagnosis of GCA. Eleven studies (including 1,578 patients) and three studies (including 149 patients) were included to evaluate ultrasound and PET/CT, respectively. The results showed a sensitivity of 86% (76–92%) and a specificity of 96% (92–98%) for cranial and large vessel ultrasound, and a sensitivity of 82% (61–93%) and a specificity of 79% (60–90%) for cranial and extracranial PET/CT. However, at the time of the meta-analysis, the authors could not identify any studies that assessed both PET/CT and ultrasound, which prevent head-to-head comparison. More recently, van Nieuwland et al. (39) included patients with suspected GCA in a nested case–control pilot study. Ultrasound, cranial and extracranial FDG-PET/CT, and cranial MRI were performed within 5 days of the initial clinical evaluation, and clinical diagnosis after 6 months of follow-up was used as gold standard. A total of 23 patients with GCA and 19 patients with suspected but undiagnosed GCA were included. The sensitivity was 69.6% (95%CI 50.4–88.8%) for ultrasound, 52.2% (95%CI 31.4–73.0%) for PET/CT and 56.5% (95%CI 35.8–77.2%) for MRI. The specificity was 100% for CDUS, FDG-PET/CT and MRI.

Another advantage of PET/CT is the ability to detect other diagnoses of interest. Firstly, PET/CT could detect neoplasms or infections that may mimic GCA. Secondly, polymyalgia rheumatica (PMR), a rheumatic disease that is often associated with GCA, can be confirmed or excluded by PET/CT. In PMR, PET/CT shows high FDG uptake in the scapula and pelvic girdles, and also in the lumbar and cervical interspinous bursae. Thirdly, PET/CT could aid the differential diagnosis of inflammatory rheumatic diseases occurring in the same age group, such as elderly-onset rheumatoid arthritis (EORA), spondyloarthropathies, crystal-induced arthropathies or remitting seronegative symmetrical synovitis with pitting oedema (RS3PE), by showing typical patterns of each disease (40).

Finally, in the case of large-vessel GCA (LV-GCA), a specific subset of GCA usually revealed by nonspecific symptoms (fatigue, fever, weight loss) and in the absence of typical signs of cranial GCA, PET/CT may be the only test that can diagnose GCA by showing vascular FDG uptake in the aorta and its main branches (39, 40).

## Prognostic accuracy

### FDG uptake evolution during follow-up

Some studies have focused on the evolution of vascular FDG uptake on therapy (mainly with glucocorticoids) by performing repeated PET/CT during follow-up. These studies showed that vascular FDG uptake decreases significantly, especially after 8 months of follow-up (41, 42), and this metabolic regression generally correlates with clinical and biological improvement (24, 43). However, other studies have observed persistent vascular uptake in patients in clinical and biological remission, defined by the absence of clinical signs and normal C-reactive protein (CRP) and/or erythrocyte sedimentation rate (ESR) (44). For example, about 80% of GCA patients who are in remission still have significant vascular uptake on PET-CT (45). In addition, Prieto-Pena et al. (46) reported a significant reduction in vascular FDG uptake in 30 LV-GCA patients followed for 10.8 ± 3.7 months, but less than one third achieved complete normalization of vascular uptake. Some authors have hypothesized that the persistence of low-grade vascular uptake may reflect smouldering inflammation or post-inflammatory vascular remodeling (45). Moreover, thoracic aortic histopathology from aortic surgery revealed active aortitis in most GCA patients despite clinical remission several years after GCA diagnosis, lending credence to the hypothesis of smouldering vasculitis persisting in patients in clinical and biological remission (47). Therefore, the value of follow-up PET/CT to predict the risk of relapse or the occurrence of aortic complication is questionable.

### PET/CT for predicting relapse

Some studies have focused on the risk of subsequent relapse in relation to persistent FDG uptake on repeat PET/CT (Table 2). Only the study by Grayson et al. (46) suggests that the value of PETVAS can be used to predict the risk of relapse during follow-up. In this study, the authors prospectively analysed patients with Takayasu’s arteritis (*n* = 26) and GCA (*n* = 30) who underwent serial PET/CT every 6 months. A total of 170 PET/CT from 56 patients with LV-GCA were analysed. PETVAS ≥20 during follow-up was associated with an increased risk of recurrence compared to patients with PETVAS <20 (55% vs. 11% of relapse, *p* = 0.003). Interpreting the study may be challenging. Firstly, patients with GCA and Takayasu’s arteritis were included. Secondly, the 30 patients with GCA were enrolled 2.6 +/− 2.7 years after diagnosis. Therefore, the patients included may have been more refractory than usual patients and at higher risk of relapse. This may explain why some of them had a PETVAS ≥20 points during follow-up, which is particularly high.

**Table 2:** Summary of studies assessing the prognostic value of PET/CT for subsequent relapse.

Studies	Design	Population	PET/CT	Results for predicting relapse
Blockmans et al. (26)	Prospective	35 GCA patients with PET/CT performed at diagnosis	TVS calculated from PET/CT performed at diagnosis, then at 3 and 6 months if the previous PET/CT showed vascular FDG uptake	Relapse versus no relapse, mean (SD): TVS at diagnosis: 5.2 (5.0) versus 7.5 (7.3), *p* = NS TVS at 3 months: 1.8 (2.0) versus 3.3 (4.3), *p* = NS TVS at 6 months: 2.8 (3.7) versus 4.8 (3.6), *p* = NS
Grayson et al. (25)	Prospective	56 LVV patients (30 with GCA and 26 with Takayasu)	PETVAS calculated from PET/CT performed at six-month intervals in patients in clinical remission	More frequent relapse in patients with PETVAS >20 (45% versus 11%, *p* = 0.03)
Sammel et al. (27)	Prospective	21 consecutive GCA patients who had PET/CT at diagnosis	TVS computed from PET/CT at diagnosis and after 6 months of follow-up	7 out of 12 (58%) patients with a TVS ≥ 10 at diagnosis relapsed compared with 5/9 (56%) with a TVS < 10
Galli et al. (47)	Retrospective	100 LVV patients (51 with GCA and 49 with Takayasu) who underwent at least one PET/CT (81 patients included in the prognostic analysis)	PETVAS computed from PET/CT performed during clinical remission with at least 6 months of follow-up	PETVAS not associated with subsequent relapses [age- and sex-adjusted HR 1.04 (95% CI 0.97, 1.11)]. AUC PETVAS in predicting subsequent relapses = 0.60 (95% CI 0.50, 0.69)
Hemmig et al. (49)	Prospective	40 GCA patients, but 25 patients included in the prognosis analysis (patients in clinical remission with PET/CT performed at treatment stop)	PET/CT positive if SUVmax artery/liver ratio > 1 for the supra-aortic region and > 1.3 for the aorta and femoral region	PET/CT positive: 4/6 (66.7%) patients who relapsed and 8/19 (42.1%) patients who remained in remission after 4 months of follow-up (*p* = 0.378)
Billet et al. (48)	Retrospective	65 patients with LVV-GCA diagnosed on PET/CT who underwent a second PET/CT after 3 to 12 months of follow-up	TVS and PETVAS calculated from the first PET/CT and second PET/CT in 55 patients in clinical and biological remission	Time-dependent ROC curves: All AUCs close to 0.5 for TVS and PETVAS calculated at first PET/CT and second PET/CT after different follow-up time

Billet et al. (48) included 55 patients with LV-GCA who underwent 2 PET/CT during the course of the disease (the first at diagnosis and the second 3–12 months later) and who were in clinical and biological remission at the time of the second PET/CT. Only 4/55 (7%) patients had a PETVAS >20 at the time of the second PET/CT. All AUROCs calculated from the time-dependent ROC curves up to 2 years after the second PET/CT were close to 0.5 for both scores (TVS and PETVAS), which means poor discriminatory power to predict relapse. However, this study also has several limitations. Firstly, patients were recruited between 2009 and 2020 in different centres with different PET/CT techniques and resolutions. Therefore, the study’s retrospective nature precluded systematic, centralized double-reading of all PET/CT images. Finally, the clinician prescribing the second PET/CT was aware of the imaging results, which may have influenced subsequent treatment decisions and relapse risk.

The study by Hemmig et al. (49) aimed to investigate the value of PET/CT and MRI in predicting relapse after stopping treatment in patients with LV-GCA (25 patients underwent PET/CT and 15 underwent MRI). A relapse occurred in 11/40 patients (27.5%) after 4 months of follow-up (time to relapse 1.9 months, IQR 1.4–3.3). Patients experiencing a relapse had no more active vasculitis on MRI and/or PET/CT (54.5% versus 58.6%, *p* = 1.0). These results are consistent with other studies detailed in Table 2, which often included patients with GCA and Takayasu’s arteritis and calculated TVS (26, 27), PETVAS or both (41).

In summary, PET/CT does not appear to predict relapse and may not be suitable for guiding treatment decisions in patients with LV-GCA in clinical remission.

### PET/CT for predicting vascular complications

Large-vessel involvement is known to be associated with an increased risk of vascular complications, particularly aortic dilatation in GCA (43). Therefore, the guidelines specify the need to monitor for structural damage, particularly at sites of previous vascular inflammation (48). This recommendation is supported by several studies.

First, the one of Quinn et al., who reported that in 32 GCA and 28 TAK patients, 80% of vascular territories with significant FDG uptake at baseline developed stenosis or aneurysms during follow-up (50). Then Blockmans et al. (51) also showed in 46 patients with a positive GCA biopsy who underwent PET/CT at diagnosis and a CT scan of the aorta during follow-up with a delay of 46.7 (29.9) months [mean (SD)] that increased FDG uptake was associated with a significantly larger diameter of the ascending and descending aorta and a significantly larger volume of the thoracic aorta. Along this line, Muratore et al. (52) reported that aortic FDG uptake grade 3 at diagnosis was associated with an increased risk of aortic dilatation compared with aortic FDG uptake ≤2. Retrospective data from the French cohort involving 549 GCA patients confirmed the results by showing that in LV-GCA, aortic dilatation occurred in a previously inflamed segment in 94% of cases (51).

More recently, Moreel et al. (53) included 106 GCA patients who had undergone PET/CT at diagnosis, within 3 days of starting glucocorticoid therapy, and who were followed by performing annual CT scans of the aorta over a ten-year period. The TVS at diagnosis was associated with a greater annual increase in thoracic aortic diameter and volume. A positive PET/CT at diagnosis was associated with a higher risk of thoracic aortic aneurysm [adjusted hazard ratio = 10.24 (CI 95%: 1.25 to 83.3)]. The authors concluded that the intensity and extent of the initial inflammation determine the risk of subsequent aortic dilatation, as no association was observed between the development of thoracic aortic aneurysm and treatment regimen or relapse rate (53). Blockmans et al. (54) performed a post-hoc analysis of this study, including 52/106 patients who had at least one further PET/CT during follow-up. A total of 88 PET/CT were analysed during follow-up, 55 during relapse and 33 during remission. Overall, 9/10 patients with thoracic aortic aneurysms had a positive PET/CT both at diagnosis and during follow-up. However, the authors emphasize that no conclusions can be drawn about FDG uptake in remission because most patients underwent repeat PET/CT during a relapse (54). Therefore, the hypothesis that persistent aortic inflammation may contribute to the development of thoracic aortic aneurysms in GCA contrasts with the lack of association between thoracic aneurysm occurrence and treatment regimen or relapse rate shown in the first part of this study (53).

In conclusion, the results of numerous studies converge on the fact that large-vessel vascular FDG uptake at GCA diagnosis is associated with an increased risk of vascular complications (mainly dilatation and aneurysm) during follow-up. Whether persistent smouldering vascular inflammation or post-inflammatory vascular remodeling is responsible for the development of aortic aneurysms is still unclear.

## Cranial PET/CT

Assessment of the cranial arteries (including temporal, occipital, maxillary and vertebral arteries) to diagnose GCA was not part of the original 2018 EULAR recommendations due to a lack of sufficient data (42). Following the publication of several studies evaluating cranial PET/CT (12–15), the updated recommendations include PET/CT alongside MRI as an alternative to ultrasonography for the examination of cranial arteries (46). Comparing these studies is challenging because different criteria were used to define a positive PET/CT and the gold standard diagnosis of GCA.

Two prospective studies by Sammel et al. (48) and Thibault et al. (15) used the clinical diagnosis as the gold standard, based on the absence of an alternative diagnosis and a favorable outcome with glucocorticoid treatment after 6 months of follow-up. Sammel et al. (48) considered the PET/CT to be positive based on a qualitative subjective evaluation of the cranial and thoracic segments. Thibault et al. (15) considered the PET/CT to be positive if at least one cranial segment had a visual grading ≥2 compared to liver FDG uptake. In the studies by Nienhuis et al. (49) and Nielsen et al. (53), patients with metastatic melanoma were used as a control group. Nienhuis et al. (55) included GCA cases with a positive TAB and Nielsen et al. (53) included GCA cases that met the ACR criteria confirmed after 6 months of follow-up. In these two case–control studies, the PET/CT was defined as positive if at least one cranial segment had a higher FDG uptake than the surrounding tissue.

The two prospective studies showed sensitivity of 71 and 73.3% and specificity of 91 and 97.2% for Sammel et al. (12) and Thibault et al. (15), respectively (54). An advantage of the study by Thibault et al. (15) was the combination of cranial PET/CT with extracranial PET/CT in a single examination. The combination of the two examinations optimized sensitivity (73.3% for cranial PET/CT, 66.7% for extracranial PET/CT and 80% for the combination) at the expense of specificity (97.2% for cranial PET/CT, 80.6% for extracranial PET/CT and 77.8% for the combination).

In conclusion, the advantage of cranial PET/CT is that it increases diagnostic sensitivity when combined with extracranial PET/CT. In addition to the temporal arteries, other cranial vessels such as the vertebral, maxillary or occipital arteries can also be studied. The correlation between the involvement of certain arterial segments and the risk of ischemic complications, for example between vertebral arteries and stroke, still requires further research. Table 3 summarises the studies’ characteristics.

**Table 3:** Summary of studies assessing PET/CT for cranial arteries in GCA.

	Sammel et al. (11)	Thibault et al. (15)	Nienhuis et al. (12)	Nielssen et al. (13)
Design	Prospective		Retrospective/Case–control	
Population	Clinical suspicion of GCA		Control group: PET/CT for follow-up of metastatic melanoma	
	64 patients, including 21 with GCA (12 positive TAB)	51 patients, including 15 with GCA (10 positive TAB)	48 patients (24 biopsy proven GCA and 24 controls)	88 patients (44 GCA including 35 with positive TAB and 44 controls)
Gold standard	Clinical diagnosis retained after at least 6 months of follow-up without alternative diagnosis		GCA confirmed by a positive TAB	GCA defined according to ACR 1990 criteria
Definition of positive PET/CT	Qualitative subjective assessment of the cranial and thoracic segments	At least one cranial segment with a visual grade ≥ 2 (≥ hepatic fixation)	At least one cranial segment with FDG uptake > surrounding tissue	A least one cranial segment (excluding occipital) with FDG uptake > surrounding tissue
Sensitivity	71% [48–89%]	73.3% [51–96%]	83% [64–93%]	82% [67–92%]
Specificity	91% [78–97%]	97.2% [92–103%]	75% [55–88%]	100% [92–100%]
Positive predictive value	79% [54–94%]	91.7% [76–107%]	Not relevant due to case–control design	
Negative predictive value	87% [73–95%]	89.7% [80–99%]		

## Perspectives

How PET/CT involvement and the extent of inflammation might guide treatment remains uncertain. Therefore, we believe that prospective evaluation of PET/CT in GCA is needed. This is especially true in clinical trials evaluating immunosuppressive therapy, where data on PET/CT assessment are lacking. In addition, the management of patients with GCA may benefit from the development and evaluation of new technologies. Examples include PET/MRI and new tracers that target the somatostatin receptor.

Combining FDG-PET with MRI may allow more precise anatomical localization of PET tracer uptake and better characterization of the inflamed arterial wall (56), while reducing radiation exposure (57). However, availability is poorer than with PET/CT and no prospective study has investigated the diagnostic performance of FDG-PET/MRI. Laurent et al. (58) defined three different patterns according to the positivity of MRI and/or PET in 13 retrospectively recruited patients with LVV who underwent 18 PET/MRIs at different follow-up times. The “inflammatory” pattern was defined as positive PET (visual grading = 3) and abnormal MRI (stenosis and/or wall thickening), the “fibrous” pattern as negative PET (visual grading = 1 or 2) and abnormal MRI (stenosis and/or wall thickening), and the “normal” pattern when both PET and MRI are negative. In a retrospective study, 14 patients with aortitis defined by PET/CT as the gold standard (11 GCA and 3 Takayasu patients) were compared with 14 control patients without aortitis (59). The sensitivity and specificity of PET/MRI were 85.7 and 100%, respectively. Sensitivity limitations were observed in the thoracic part of the aorta due to motion artefacts.

False-positive results from FDG PET/CT may be due to the metabolic activity of atherosclerosis, which is sometimes difficult to distinguish from persistent smoldering vascular inflammation or vascular remodeling, calling for the development of new, more specific radiotracers. Targeting the somatostatin receptor expressed by inflammatory macrophages, which play a major role in the pathophysiology of GCA, is an interesting prospect that could meet this need. Among these, somatostatin receptor PET/MRI using ^68^Ga-DOTATATE or ^8^F-FET-βAG-TOCA are candidates for more specific evaluation of large vessel vasculitis (60). In this prospective study, Ćorović et al. (60) compared 61 patients, including 27 with LVV (GCA = 13, Takayasu = 13, unspecified LVV = 1), 25 with recent atherosclerotic myocardial infarction and 9 patients with cancer. PET/MRI with ^68^Ga-DOTATATE and ^18^F-FET-bAG-TOCA discriminated active LVV from inactive LVV and active LVV from athrosclerosis with high diagnostic accuracy (AUROC = 0.89 and AUROC = 0.86, respectively).

## Conclusion

PET/CT imaging has high diagnostic accuracy in GCA by demonstrating transmural vascular inflammation in large vessels. Recently, sensitivity has been improved by the ability to detect vascular FDG uptake in cranial arteries. LVV detected by PET/CT correlates with disease activity and could predict vascular complications such as aneurysms, suggesting that assessment of vascular damage by morphologic imaging during follow-up is warranted in these patients. However, PET/CT has several limitations. First, significant vascular FDG uptake may remain in some patients in remission on therapy. It is unclear whether this FDG uptake is due to persistent smouldering vascular inflammation or post-inflammatory vascular remodeling. In particular, the persistence of this FDG uptake does not appear to be predictive of future relapse and therefore should not be used to guide treatment decisions in patients in clinical remission. Secondly, the main limitation to the generalization of PET/CT is its availability in most centres less than 72 h after the introduction of glucocorticoids, after which the diagnostic accuracy decreases significantly. This limitation is very problematic because glucocorticoids must be started early after suspicions because of the risk of ophthalmological complications and blindness.
